# Workplace support for employees with cancer

**DOI:** 10.3747/co.v16i5.381

**Published:** 2009-09

**Authors:** B. Nowrouzi, N. Lightfoot, K. Cote, R. Watson

**Affiliations:** * Master of Public Health Program, Lakehead University, Thunder Bay, ON; † Centre for Addiction and Mental Health, Toronto, ON; ‡ School of Rural and Northern Health, Laurentian University, Sudbury, ON; § Department of Public Health Sciences, University of Toronto, Toronto, ON; || Northern Ontario School of Medicine, East Campus, Sudbury, ON; # Sudbury Regional Hospital, Sudbury, ON

**Keywords:** Workplace support, return to work, employees with cancer, employer assistance

## Abstract

**Objective:**

The aim of the present study was to survey human resources personnel about how their northeastern Ontario workplaces assist employees with cancer.

**Study Design and Setting:**

This cross-sectional study was conducted from December 2007 to April 2008. Surveys were sent to 255 workplaces in northeastern Ontario with 25 or more employees, and 101 workplaces responded (39.6% response rate). Logistic regression modelling was used to identify factors associated with more or less workplace support. More or less workplace support was defined by provision of paid time to employees with medical appointments *and* an offer of a return-to-work meeting *and* reduced hours for employees with cancer. Factors considered in the model included organization size, geographic location (urban, rural), and workplace type (private sector, public sector).

**Results:**

Most of the human resources staff who completed the surveys were women (67.4%), and respondents ranged in age from 25 to 70 years (mean: 45.30 ± 8.10 years). Respondents reported working for organizations that ranged in size from 25 to more than 9000 employees. In the logistic regression model, large organization size [odds ratio (or): 6.97; 95% confidence interval (ci): 1.34 to 36.2] and public sector (or: 4.98; 95% ci: 1.16 to 21.3) were associated with employer assistance. Public sector employers provided assistance at a rate 5 times that of private sector employers, and large organizations (>50 employees) provided assistance at a rate 7 times that of smaller organizations.

**Conclusions:**

In the population studied, employees with cancer benefit from working in larger and public sector organizations. The data suggest a need for further support for employees with cancer in some other organizations.

## 1. INTRODUCTION

Cancer has a substantial effect on health status, depression, and overall quality of life 1–5. As early detection and more effective interventions emerge, the prevalence of cancer survivors in the population continues to increase 6. Many cancer patients resume their activities of daily living shortly after treatment. Cancer is not only an issue for the individuals and their families, but also an important issue for employers and the workplace 7. Canadian workplaces need to be aware of health and safety legislation 8 and the legal rights of workers.

Therapeutic approaches have increased not only cancer survivorship, but also the ability of affected people to work during and after treatment. However, the effect that both diagnosis and treatment have on the ability of cancer survivors to fully engage in paid work is not yet entirely understood 9. In 2004, a population-based investigation in the United States reported that, as compared with healthy controls matched on sex, age, and educational attainment, survivors had worse outcomes across all measures of burden, including work 10. Since 2000, two reviews of research related to the workplace and cancer have been published 11,12. A dearth of evidence on the effect of cancer on workplace outcomes was noted. Furthermore, more research was recommended to assess the disease-, person-, and work-related factors and the associated relationships that may have an effect on work life and return to work 13. Recent studies have focused on the effects of cancer on employment 11.

Cancer is a public health concern that will increase in importance over the next 10 years as treatments become more successful and as the population ages 14. The employment status of cancer patients and survivors has important implications for society and the labour market, for organizations, for the individuals affected, and for their economic, social, and psychological health 15. Data suggest that, in Canada, approximately 62%–84% of cancer survivors return to work after treatment; similar patterns are found in the United States and Europe 16–19. However, very little is known about specific psychosocial factors, affective continuance, and return to work 6.

Work is important for an individual’s identity and provides a social connection; it also presents a distraction and enables the person to regain a sense of normality and control 20. Return to work after a critical illness such as cancer is an important area of study. First, returning to or maintaining employment after cancer is important for a person’s quality of life, including physical and mental health 21. Earnings from employment are necessary to meet basic needs and to facilitate a return to usual life activities 19. Moreover, for many women, returning to work after a cancer diagnosis is an important measure of recovery from, and control of, the disease 8,22–25. Second, although legislation in Canada (the Employment Equity Act, 1995) protects workers against discrimination on the basis of handicap or health state, cancer survivors in the United States and Canada have reported experiencing problems in the workplace after returning to work 17,26. Problems noted have included hostility, discrimination, decreased wages, and difficulty obtaining a new job 17,26. Returning to work serves as a positive step toward the future by providing social and financial support to employees with cancer 22,25,27. Cancer patients able to fulfil social and occupational roles while undergoing active cancer treatment consider themselves to be healthy 24.

Previous studies conducted in people treated for various types of cancer have reported a variety of problems at work, including job loss 25,28,29, undesired changes in work situation 21,25,28–30, problems with co-workers 21,25,28,30,31, and diminished work capacity 18,22,28. The research has typically focused on employability statistics, adopting a health economics perspective 32,33. Currently, the focus has turned toward the employment outcomes of cancer survivors 34. Furthermore, studies have identified that, although 1 in 5 cancer survivors reported cancer-related disabilities at follow-up by telephone interview from four medical centers in Maryland and Pennsylvania, half continued to work 34.

The challenges and consequences of cancer and its treatment approaches are likely to affect, in many ways, an individual’s ability to work. These challenges and consequences include physical factors related to the disease such as disfigurement or pain following surgery 33,35, fatigue 11, and decreased cognitive functioning 36,37. A Fatigue Coalition study conducted telephone interviews with 379 randomly selected participants with a previous history of cancer in the United States. Of the 177 who were working, 75% had made changes in their employment status as a result of fatigue, 71% had missed 1 or more days of work per week, 34% had reduced their hours or accepted fewer responsibilities, 23% had gone on disability, and 28% had stopped working 38–40. Many of these side effects and consequences of cancer and associated interventions may be enduring and may last for many years post treatment. Many of these factors are also seen in other chronic illnesses 41.

Most studies that address cancer and work outcomes have focused on the likelihood and timeliness of work return 42. A recent literature review by Spelten *et al.* 11 summarized fourteen studies and identified several features of the cancer, the job, and the person that influence work outcomes. Those authors also highlighted the methodologic and conceptual limitations of the research to date. In particular, the examined studies did not use similar measures of work return, were often methodologically weak, and tended to study highly selected samples of cancer survivors with specific cancer sites 42.

Previous studies have shown that the ability of cancer survivors to continue employment with employer support in the workplace appears optimistic 43. Research conducted using telephone interviews (participants were interviewed by telephone 12 and 18 months after diagnosis) of women with breast cancer from the Metropolitan Detroit Cancer Surveillance System revealed that 80% had returned to work during a period of 18 months after their cancer diagnosis 44. As compared with women never diagnosed with cancer, survivors were slightly more likely not to be working 3 years after diagnosis 12. However, in a cross-sectional mailed survey about the effect of illness on their vocational status, answered by 378 women who had survived breast cancer without recurrence for at least 2 years, more than 40% said that cancer had altered their priorities or their progress at work 45.

Currently, a full understanding has not been achieved concerning how cancer patients perceive their ability to work or the adjustments that are required to facilitate work during and following treatment. Studies that have attempted to address these concerns continue to be hampered by small sample sizes and a lack of control for cancer site. In-depth studies have largely been restricted to clinical case studies and qualitative research 16.

Serious illness in the workplace raises complicated issues for employers, including right to privacy, concerns of fellow workers, and accommodation and productivity. As the number of cancer survivors increases, empirical data on their work experience is growing. Quantitative studies using questionnaires have suggested that a change of job or employer, early retirement, unemployment, and lowered income are common among cancer patients 19,46. Qualitative studies have indicated that women with breast cancer returning to work experience physical fatigue, demotion, conflict with employers and co-workers, personal changes in attitude toward their job, and unwanted job responsibilities 17,47. Little information is available about the employment changes of cancer patients and the factors related to those changes 48. Although studies have been conducted regarding social support for cancer patients (mostly using qualitative methodology), the importance of support from work life is unclear.

Cancer places major social, economic, and psychological burdens on individuals and their relationships (personal and professional). Research indicates that there is very little evidence concerning how employers assist employees with cancer.

## 2. STUDY DESIGN AND SETTING

Our study used a non-experimental, cross-sectional survey design. Human resources personnel in workplaces with at least 25 employees in northeastern Ontario were invited to participate in the study. A total of 255 paper questionnaires were mailed to human resources personnel at Ontario businesses listed in the Canadian Business Directory. An online version of the questionnaire was also made available. A reminder letter was mailed 2 weeks after the initial study package, and additional follow-up consisted of telephone calls at 4 weeks and 6 weeks after the first mailing. At week 8, a reminder e-mail message was sent to workplaces that had not replied.

In total, 101 responses to the survey were received (39.6% response rate), either online or in paper form.

### 2.1 Measures

#### 2.1.1 Employer Assistance

Employer assistance was defined as paid time for medical appointments, *and* an offer of a return-to-work meeting, *and* reduced hours for employees with cancer. Evidence has shown that paid time for medical appointments is associated with return to work for employees with cancer 9. A return-to-work meeting showed significance (*p* = 0.006) in a Fisher exact two-tailed test analysis.

#### 2.1.2 Number of Employees in the Organization

Respondents were asked to report the number of employees at their organization. This variable was partitioned into two categories: 25–49 employees and 50 or more employees. The data were divided this way because the division allowed for half the sample to be represented in each category.

#### 2.1.3 Urban or Rural Centre

We separated the communities of northeastern Ontario into urban and rural categories. “Urban” was defined as a centre with 10,000 or more people, and “rural” was defined as a center with fewer than 10,000 people 49,50. Populations for the communities were obtained from Statistics Canada community profiles for 2006.

#### 2.1.4 Private and Public Sectors

Employers were separated into public and private sector categories based on participant responses. “Private sector” included manufacturing, insurance, and retail businesses. “Public sector” included governmental bodies, education boards, and non-profit organizations.

#### 2.1.5 Types of Accommodations

The “job sharing” classification included the sharing or division of job duties and responsibilities of an employee with cancer, and “reduction in hours worked” encapsulated a reduction in the number of hours worked by the employee with cancer. “Telecommuting” was classified as working from home and is part of teleworking (home and regional centres are the two main types of telecommuting). “Additional breaks or rest periods” was defined as provision of breaks as needed by employees in addition to the breaks normally scheduled during a typical work period. “Adjustments in the physical environment” was identified as employer-supported modifications to the physical setting of an employee’s workspace, such as ergonomic office assessment or job-site analysis. Other accommodations were “paid time for medical appointments” and offer of a “return-to-work meeting”—an organized meeting with a return-to-work representative, the employer, and the employee with cancer.

#### 2.1.6 Employer’s Perspective

“Employees’ work responsibilities” was based on the opinion of the responding human resources professional regarding whether employees with cancer can fulfil their work responsibilities and deal with their illness at the same time. “Tracking of employees with cancer” was defined as whether an organization officially tracks the number of employees with cancer or those that return to work after treatment.

## 3. RESULTS

### 3.1 Characteristics of the Study Population

Descriptive and univariate analyses were conducted to describe the characteristics of the participants. Of the 101 responses, 41 consisted of paper questionnaires, and 60, of questionnaires completed online. Most of the human resources staff who completed the surveys were women (67.4%), and respondents ranged in age from 25 to 70 years (mean: 45.30 ± 8.10 years). Respondents reported working for organizations that ranged in size from 25 to more than 9000 employees. The human resources directors had, on average, 11 years of experience (mean: 11.31 ± 8.31 years). With regard to type of workplace, 65.3% of respondents (*n* = 101) were reporting on manufacturing workplaces, and 11.9%, on other areas of the private sector. Conversely, 15.8% of respondents indicated that they were reporting for a public sector workplace; 6.93% classified their workplace as “Other.” Table I provides details the characteristics of the respondents and the workplaces.

### 3.2 Factors Relating to How Employers Assist Employees with Cancer

The factors associated with employer assistance to employees with cancer were subjected to univariate and multivariate logistic regression analyses. The predictor variables in the logistic model included the number of employees in an organization, the setting (urban or rural) of an organization, and the sector (private or public) represented by an organization (see Table II).

When paid time for medical appointments, reduction in work hours, and provision of a return-to-work meeting were considered, employers with more than 50 workers [odds ratio (or): 6.97; 95% confidence interval (ci): 1.34 to 36.2] and employers in the public sector were significant (or: 4.98; 95% ci: 1.16 to 21.3).

## 4. DISCUSSION

The purpose of the present study was to increase understanding of how employers assist employees with cancer. The ramifications of cancer are not confined to the workplace; they are widespread, affecting personal and professional relationships alike 51. Moreover, cancer affects a worker’s physical and mental well-being and cognitive functioning 11,19,33,35–37,52. It is important that workers with cancer and their employers are aware of the effect that the symptoms of cancer and that cancer treatment have on the employee, and that they discuss the resulting changes to the work and the job requirements.

In examining organizations that offered accommodations to employees with cancer (those reported to be significant), public sector employers offered assistance in greater proportion. Our finding concerning paid time for medical appointments accords with the 2007 study by Pryce *et al.* 9 in that such time is a significant factor in predicting return to work after cancer. In terms of organization size, organizations with more than 50 employees provided more assistance in certain areas—for example, a return-to-work meeting, paid time for medical appointments, and reduction in work hours—than did smaller businesses. That finding may not be surprising, given that larger employers have greater access to financial and other types of resources. It is encouraging that employers are offering resources and assistances to their employees, but a greater emphasis should be placed on identifying the services that are requested by or essential to employees with cancer. Such identification is challenged by the individualistic nature of the disease (for example, no two people are alike, and neither are their cancer outcomes) and the consequences for both the employer and the employee.

### 4.1 Limitations and Potential Biases

Our research has some limitations. At 39.6%, the survey response rate was lower than expected, despite the systematic follow-up procedures: that is, the reminder letter, the two reminder telephone calls, and the two e-mail messages. The response rate was comparable to that in the employee survey by Pryce *et al.* 9 of factors related to return to work. More responses were provided online because the questionnaire was readily available to late responders or to those who no longer had a paper copy.

Response rates likely depend more on the population sampled than on any other factor 53. Standardized questionnaires delivered online and on paper have produced mixed results 54. However, considering that the study offered participants no remuneration, obtaining a higher response rate becomes highly challenging. Compensating the participants may have increased the number of participants in the present study. The human resources professionals may have been too preoccupied with work responsibilities to participate in the study. Research has shown that a low response rate alone does not necessarily indicate bias. When participant characteristics are representative of non-responders, low return rates are not biasing 55,56. However, the response rate achieved here limits both the internal and external validity of the study.

Our study used a quantitative approach ultimately to describe how employers on a large scale are offering assistance to their workers with cancer. Almost half the employers did not believe that employees could manage their work responsibilities simultaneously with their illness. Some employers may perceive that employee limitations are more significant than those limitations are in reality; others may perceive that employees can work harder than those employees can work in reality. Nearly half the employers (47.5%) believed that employees with cancer could simultaneously fulfil their work obligations and manage their illness. Some employers may view worker limitations as more significant than they actually are. Conversely, other employers may minimize the effect of an employee’s illness and have higher expectations. These expectations can have a serious effect on the individual’s work performance and professional relationships—not only with the employer, but also with coworkers.

The representativeness of cases in this study is of potential concern. The sample population was confined to northeastern Ontario. Therefore, the results cannot be generalized beyond northeastern Ontario. Secondly, participants were selected from the Canadian Business Directory. Not all northeastern Ontario businesses are listed in the directory. Furthermore, 15 study packages were returned because they were undeliverable—for example, for not having the current address of the business. Also, questionnaire respondents may be more motivated, interested, or inclined to help with the study than non-respondents are. In Canada, 75% of human resource specialists are female 57, and our sample may be an adequate representation of this group. Most of the respondents (67.4%) to our survey were women, which may suggest that women respondents were underrepresented in our sample. Older participants constituted a greater proportion of the sample (19.6%) than appears in the larger group of human resource specialists as reported by Service Canada 57. This finding could indicate that an important group (younger respondents) is being underrepresented, and another (older respondents) is being overrepresented. Finally, the study surveyed organizations with 25 or more employees, thus excluding the responses of smaller organizations.

### 4.2 Future Research

The low response rate may suggest that a qualitative approach with emphasis on focus groups and key informant interviews may be beneficial in identifying themes important to the question of how employers help their employees. The interrelated nature of cancer-related factors and their effect on return to work makes it challenging to identify potential relationships with the outcome measure 11.

Numerous studies have shown that, as compared with a cancer-free population, cancer survivors have a lower probability of being employed 51. Pryce *et al.* 9 suggest further research to examine and understand psychosocial predictors related to work. It is plausible that some of these factors may be related to how employers offer assistance during this tumultuous period in the employee’s personal and professional life. It would be beneficial to understand the relationship between employer support and return to work and the effect of employer support on workload, on support for colleagues of employees with cancer, and on productivity. Qualitative studies could also use narratives and focus groups to identify themes within the organization at various corporate levels. Conversely, further quantitative studies could target human resource professionals in other jurisdictions or use a larger sample size to possibly validate the results of the present study. A mixed-methods approach may be beneficial in combining a survey sent to employers with interviews.

Given the hierarchical nature of businesses, future research could use a hierarchical approach to further examine the views both of employers and of employees within an organization from a particular region 58. In organizational studies, investigators might examine how workplace characteristics such as providing additional supports and resources (for example, paid time for medical appointments, reduction in work hours, and a return-to-work meeting) influence productivity of employees with cancer 59. Employees and organizations are both units in the analysis: variables are measured at both levels. As a result, the data has a hierarchical structure, with individual workers nested within workplaces. Hierarchical linear modelling has been used in public health, psychology, and education to tackle some common problems associated with multilevel data, thus advancing the understanding of the dynamic inner workings of organizations 58. Independent of the methodologic approach used, researchers need to examine the interaction between the many factors involved in the return-to-work process. Investigators should examine the importance of workplace supports for cancer survivors and those dealing with their illness while working.

### 4.3 Implications of Research for Stakeholders

Too few employers are providing sufficient support and information to employees affected by cancer. The present study showed that only a small proportion of employers have either a specific policy on managing cancer in the workplace or a generic policy on critical illness. Policies are not a universal solution to this complex problem. However, they can set out clearly for employers and employees at all levels the resources and support available within an organization and can help to ensure that individuals affected by illness are managed in a consistent manner. As well, employers should officially track the number of employees with cancer to be able to deliver services in a timely and appropriate manner and to gauge the demand for those services in the workplace.

As several respondents stated, cancer in the workplace is a sensitive, personal, and individualized issue for employees. Furthermore, its implications are widespread and often involve employers, colleagues, co-workers, and personal relationships with family and friends. During a period of great uncertainty, change and conflicting emotional approaches to an issue that is not standard, employers can provide a wealth of resources and become a beacon of stability to employees with cancer. Concurrently, employers are in an ideal position to facilitate flexibility and an understanding of employees’ apprehensions or concerns regarding their ability to return to work, because a decrease in wages can pose a significant financial burden. Equally important is the implementation of a cancer policy that is relevant and available to employees. Our study indicates that this area appears to be one in which organizations may need to invest more resources.

The present research has the potential to offer important information to four different groups. First, clinicians from primary to tertiary care may be able to use the results of this study to enhance the level of care they provide to patients. They may also use the findings to develop better relationships that foster timely communication with the employee’s workplace. Research has shown that, the greater the level of involvement of health care professionals in the return-to-work process, the more likely it is that the employee will return to work sooner.

Second, this novel and under-investigated research topic deserves increased attention from researchers. It is especially relevant when considering the pervasiveness of cancer in Canadian society. Some research has emerged from the United States and the United Kingdom, but very little Canadian literature addresses this topic.

Third, for human resources professionals and employers, this research may help in raising some of the issues that are important to them in dealing with an employee with cancer.

Finally, although employees were not directly involved in the study, we believe that they could reach a greater understanding of the employer’s role in the process and the factors that affect their chances to resume their occupation.

## 5. CONCLUSIONS

The present study highlights the importance of cancer management for employers and their employees. Additional research may indicate that when work adjustments are tailored to meet the needs of employees with cancer, those employees are most likely to continue working or to return to work. That understanding may be part of future research that allows for a deeper level of collaboration between all stakeholders.

## Figures and Tables

**TABLE I:** Demographics of study participants

Characteristic	Total^a^ (*n*=92)	Men (*n*=30)	Women (*n*=62)
Age			
<35 years	9.8	10.0	9.7
36–45 years	37.0	16.7	46.8
46–55 years	33.7	46.7	27.4
≥56 years	19.6	26.7	16.1
Years of experience in hr			
≤10 years	54.3	46.7	58.1
11–20 years	30.4	26.7	32.3
>20 years	15.2	26.6	9.7
Size of workforce			
25–50 employees	50.0	60.0	45.2
≥51 employees	50.0	40.0	54.8
Type of workplace^b^			
Manufacturing	64.1	76.7	58.1
Private sector	12.0	10.0	12.9
Public sector	15.2	6.7	19.4
Non-profit organization	2.2	0	3.2
Other	6.5	6.7	6.5

a Responses numbered 92 because of missing values.

b Examples of workplace by category: Manufacturing—automobile, steel; Private sector—information technology, retail; Public sector —government, education; Non-profit organization—YMCA, Salvation Army.

hr = human resources.

**TABLE II:** Adjusted odds ratio estimates and approximate 95% confidence intervals (ci) for employer assistance of employees with cancer (*n* = 100)

Characteristic	Odds ratio	95% ci
Number of employees^a^	6.97	1.34 to 36.21
Private versus public sector^a^	4.98	1.16 to 21.3
Urban versus rural	5.05	0.91 to 28.00

a *p* < 0.05 (overall logistic regression based on all predictor variables).

## References

[1] Bodurka–Bevers D Basen–Engquist K Carmack CL Depression, anxiety, and quality of life in patients with epithelial ovarian cancer Gynecol Oncol 2000 78 pt 1 302 8 10985884 10.1006/gyno.2000.5908

[2] Crom DB Chathaway DK Tolley EA Mulhern RK Hudson MM Health status and health-related quality of life in long-term adult survivors of pediatric solid tumors Int J Cancer Suppl 1999 12 25 31 10679867 10.1002/(sici)1097-0215(1999)83:12+<25::aid-ijc6>3.0.co;2-q

[3] Ganz PA Schag CC Heinrich RL Barofsky I Information about work from studies with the Cancer Inventory of Problem Situations (cips) Work and Illness: The Cancer Patient New York Praeger 1989 117 32

[4] Hopwood P Stephens RJ Depression in patients with lung cancer: prevalence and risk factors derived from quality-of-life data J Clin Oncol 2000 18 893 903 10673533 10.1200/JCO.2000.18.4.893

[5] Ramsey SD Andersen MR Etzioni R Quality of life in survivors of colorectal carcinoma Cancer 2000 88 1294 303 10717609

[6] Feuerstein M Cancer survivorship and work J Occup Rehabil 2005 15 1 2 15794491 10.1007/s10926-005-0868-x

[7] Schultz PN Beck ML Stava C Sellin RV Cancer survivors. Work related issues AAOHN J 2002 50 220 6 12033089

[8] Lightfoot N Dumont J Conlon M Collection and retention of demographic, medical, and occupational information in northeastern Ontario workplaces Chronic Dis Can 2003 24 17 26 12757632

[9] Pryce J Munir F Haslam C Cancer survivorship and work: symptoms, supervisor response, co-worker disclosure and work adjustment J Occup Rehabil 2007 17 83 92 17318459 10.1007/s10926-006-9040-5

[10] Yabroff KR Lawrence WF Clauser S Davis WW Brown ML Burden of illness in cancer survivors: findings from a population-based national sample J Natl Cancer Inst 2004 96 1322 30 15339970 10.1093/jnci/djh255

[11] Spelten ER Sprangers MA Verbeek JH Factors reported to influence the return to work of cancer survivors: a literature review Psychooncology 2002 11 124 31 11921328 10.1002/pon.585

[12] Steiner JF Cavender TA Main DS Bradley CJ Assessing the impact of cancer on work outcomes: what are the research needs? Cancer 2004 101 1703 11 15386303 10.1002/cncr.20564

[13] Taskila T Lindbohm ML Factors affecting cancer survivors’ employment and work ability Acta Oncol 2007 46 446 51 17497311 10.1080/02841860701355048

[14] Feuerstein M Harrington CB Recommendations for the U.S. National Occupational Research Agenda: research on cancer survivorship and musculoskeletal disorders and work disability J Occup Rehabil 2006 16 1 5 16688488 10.1007/s10926-005-9004-1

[15] Hewitt ME Greenfield S Stovall E From Cancer Patient to Cancer Survivor: Lost in Transition Washington, DC National Academies Press 2005

[16] Edwards BK Brown ML Wingo PA Annual report to the nation on the status of cancer, 1975–2002, featuring population-based trends in cancer treatment J Natl Cancer Inst 2005 97 1407 27 16204691 10.1093/jnci/dji289

[17] Maunsell E Brisson C Dubois L Lauzier S Fraser A Work problems after breast cancer: an exploratory qualitative study Psychooncology 1999 8 467 73 10607979 10.1002/(sici)1099-1611(199911/12)8:6<467::aid-pon400>3.0.co;2-p

[18] Short PF Vargo MM Responding to employment concerns of cancer survivors J Clin Oncol 2006 24 5138 41 17093276 10.1200/JCO.2006.06.6316

[19] van der Wouden JC Greaves–Otte JG Greaves J Kruyt PM van Leeuwen O van der Does E Occupational reintegration of long-term cancer survivors J Occup Med 1992 34 1084 9 1432298 10.1097/00043764-199211000-00010

[20] Peteet JR Cancer and the meaning of work Gen Hosp Psychiatry 2000 22 200 5 10880715 10.1016/s0163-8343(00)00076-1

[21] Anderson NB Armstead CA Toward understanding the association of socioeconomic status and health: a new challenge for the biopsychosocial approach Psychosom Med 1995 57 213 25 7652122 10.1097/00006842-199505000-00003

[22] Clark JC Landis LL Reintegration and maintenance of employees with breast cancer in the workplace AAOHN J 1989 37 186 93 2712882

[23] Holland J Special problems of cancer patients returning to work Trans Assoc Life Insur Med Dir Am 1986 69 87 94 3739138

[24] Kagawa–Singer M Redefining health: living with cancer Soc Sci Med 1993 37 295 304 8356479 10.1016/0277-9536(93)90261-2

[25] Mellette SJ The cancer patient at work CA Cancer J Clin 1985 35 360 73 3931869 10.3322/canjclin.35.6.360

[26] Feldman FL Female cancer patients and caregivers: experiences in the workplace Women Health 1986 11 137 53 3564496 10.1300/j013v11n03_10

[27] Ferrell BR Grant MM Funk B Otis–Green S Garcia N Quality of life in breast cancer survivors as identified by focus groups Psychooncology 1997 6 13 23 9126712 10.1002/(SICI)1099-1611(199703)6:1<13::AID-PON231>3.0.CO;2-S

[28] Feldman FL Barofsky I Inquiries into work experiences of recovered cancer patients: the California experience Work and Illness: The Cancer Patient New York Praeger 1989 25 47

[29] Leigh S Cancer survivorship: a first-person perspective Cancer Nurs 2006 29 suppl 12 14 16779949 10.1097/00002820-200603002-00004

[30] Steele JA Myths about cancer lead to workplace discrimination J Natl Cancer Inst 1993 85 92 3 8418312 10.1093/jnci/85.2.92

[31] Brown HG Ming TS Vocational rehabilitation of cancer patients Semin Oncol Nurs 1992 8 202 11 1523368 10.1016/0749-2081(92)90018-x

[32] Bradley CJ Bednarek HL Employment patterns of long-term cancer survivors Psychooncology 2002 11 188 98 12112479 10.1002/pon.544

[33] Chirikos TN Russell–Jacobs A Jacobsen PB Functional impairment and the economic consequences of female breast cancer Women Health 2002 36 1 20 12215000 10.1300/J013v36n01_01

[34] Short PF Vasey JJ Tunceli K Employment pathways in a large cohort of adult cancer survivors Cancer 2005 103 1292 301 15700265 10.1002/cncr.20912

[35] Cella DF Tross S Psychological adjustment to survival from Hodgkin’s disease J Consult Clin Psychol 1986 54 616 22 3771879 10.1037//0022-006x.54.5.616

[36] Ahles TA Saykin AJ Furstenberg CT Quality of life of long-term survivors of breast cancer and lymphoma treated with standard-dose chemotherapy or local therapy J Clin Oncol 2005 23 4399 405 15994149 10.1200/JCO.2005.03.343 PMC1237110

[37] Minisini A Atalay G Bottomley A Puglisi F Piccart M Biganzoli L What is the effect of systemic anticancer treatment on cognitive function? Lancet Oncol 2004 5 273 82 15120664 10.1016/S1470-2045(04)01465-2

[38] Carlson RH There’s more to fatigue than anemia Oncol Times 2001 23 128 31

[39] Curt GA Cancer fatigue: diagnosis, treatment and economic impact Oncol Economics 2000 1 29 33

[40] Curt GA Breitbart W Cella D Impact of cancer-related fatigue on the lives of patients: new findings from the Fatigue Coalition Oncologist 2000 5 353 60 11040270 10.1634/theoncologist.5-5-353

[41] Munir F Jones D Leka S Griffiths A Work limitations and employer adjustments for employees with chronic illness Int J Rehabil Res 2005 28 111 17 15900180 10.1097/00004356-200506000-00003

[42] Main DS Nowels CT Cavender TA Etschmaier M Steiner JF A qualitative study of work and work return in cancer survivors Psychooncology 2005 14 992 1004 15744780 10.1002/pon.913

[43] Baanders AN Andries F Rijken PM Dekker J Work adjustments among the chronically ill Int J Rehabil Res 2001 24 7 14 11302468 10.1097/00004356-200103000-00002

[44] Bouknight RR Bradley CJ Luo Z Correlates of return to work for breast cancer survivors J Clin Oncol 2006 24 345 53 16421415 10.1200/JCO.2004.00.4929

[45] Stewart DE Cheung AM Duff S Long-term breast cancer survivors: confidentiality, disclosure, effects on work and insurance Psychooncology 2001 10 259 63 11351378 10.1002/pon.499

[46] Abrahamsen AF Loge JH Hannisdal E Holte H Kvaloy S Socio-medical situation for long-term survivors of Hodgkin’s disease: a survey of 459 patients treated at one institution Eur J Cancer 1998 34 1865 70 10023307 10.1016/s0959-8049(98)00269-x

[47] Salander P Bergenheim AT Henriksson R How was life after treatment of a malignant brain tumour? Soc Sci Med 2000 51 589 98 10868672 10.1016/s0277-9536(00)00002-2

[48] Choi KS Kim EJ Lim JH Job loss and reemployment after a cancer diagnosis in Koreans—a prospective cohort study Psychooncology 2007 16 205 13 16894641 10.1002/pon.1054

[49] du Plessis V Beshiri R Bollman RD Clemenson H Definitions of rural Rural Small Town Can Anal Bull 2001 3 1 17 [Available online at: www.statcan.gc.ca/pub/21-006-x/21-006-x2001003-eng.pdf; cited May 21, 2009]

[50] Pong RW Pitblado JR Geographic Distribution of Physicians in Canada: Beyond How Many and Where Ottawa Canadian Institute for Health Information 2005 [Available online at: secure.cihi.ca/cihiweb/products/Geographic_Distribution_of_Physicians_FINAL_e.pdf (registration required); cited April 4, 2009]

[51] Hoffman B Cancer survivors at work: a generation of progress CA Cancer J Clin 2005 55 271 80 16166073 10.3322/canjclin.55.5.271

[52] Greenwald HP Dirks SJ Borgatta EF McCorkle R Nevitt MC Yelin EH Work disability among cancer patients Soc Sci Med 1989 29 1253 9 2532788 10.1016/0277-9536(89)90065-8

[53] Sax L Gilmartin SK Bryant AN Assessing response rates and nonresponse bias in web and paper surveys Res Higher Educ 2003 44 409 32

[54] Vallejo MA Mañanes G Isabel Comeche MA Díaz MI Comparison between administration via Internet and paper-and-pencil administration of two clinical instruments: SCL-90-R and GHQ-28 J Behav Ther Exp Psychiatry 2008 39 201 8 17573039 10.1016/j.jbtep.2007.04.001

[55] Dillman DA The design and administration of mail surveys Annu Rev Sociol 1991 17 225 49

[56] Korsnick JA Survey research Annu Rev Psychol 1999 50 537 67 15012463 10.1146/annurev.psych.50.1.537

[57] Canada, Service Canada Job Futures > Specialists in Human Resources [Web page] Gatineau, QC Service Canada 2007 [Available online at: www.jobfutures.ca/noc/1121p4.shtml; cited May 15, 2009]

[58] Todd SY Crook TR Barilla AG Hierarchical linear modeling of multilevel data J Sport Manage 2005 19 387 403

[59] Raudenbush SW Bryk AS Hierarchical Linear Models: Applications and Data Analysis Methods2nd ed Thousand Oaks, CA Sage Publications 2002

